# Moving towards deep equity, diversity, inclusivity and accessibility in simulation: a call to explore the promises and perils

**DOI:** 10.1186/s41077-024-00278-3

**Published:** 2024-02-08

**Authors:** Peter Dieckmann, Latika Nirula

**Affiliations:** 1 Copenhagen Academy for Medical Education and Simulation (CAMES), Herlev, Denmark Borgmester Ib Juuls Vej 1b, 2730; 2 Department of Quality and Health Technology, University in Stavanger, Stavanger, Norway; 3 Deparment of Public Health, Copenhagen University, Copenhagen, Denmark; 4 Li Ka Shing Knowledge Institute, Unity Health Toronto, Toronto, Canada; 5 Department of Psychiatry, University of Toronto, Toronto, Canada; 6 Centre for Faculty Development, University of Toronto, Toronto, Canada

**Keywords:** Equity, Diversity, Inclusivity, Inclusion, Accessiblity, Simulation, Healthcare

## Abstract

Principles and issues of equity, diversity, inclusivity, and accessibility (EDIA) are being explored currently in simulation designs and trainings but with limited depth, often raising more questions than answers. This editorial invites the broader healthcare simulation community to move beyond the superficial to explore more expansively and deeply these issues of EDIA within simulation. Simulation is the very environment and context from which we may confront how existing (power) structures can be dismantled and re-envisioned for more optimal redistribution of participation, power, and benefits. We can use simulation to experiment with variations of these realities, and start exploring consequences of such alternatives to benefit our broader health systems and societies. Simulation uniquely combines opportunities for experience, reflection, application and active experimentation, enabling a ripe ground for this study. In fact, it is the responsibility of simulation educators to take up this challenge, and to engage in meaningful scholarship to understand more about the impact of simulation in exploring EDIA topics. This editorial invites contributions of empirical and theoretical works that advance our collective understanding of EDIA, while also cautioning against complacency. The simulation community is urged to look inwards and also examine its own practices critically, in spite of the uncertainty, vulnerability and risks that this presents.

## Background

We were unsettled, but motivated, by a recent online discussion. The discussion centered around questions about simulation in equity, diversity, inclusivity, and accessibility (EDIA): Why is simulation in this space of EDIA training? What unique affordances does it offer? What is being simulated (and what is not), by whom, for whom? Who might the simulation help, and who might it harm? And perhaps most importantly, “What might be explicitly or implicitly perpetuated and reinforced – or ignored?” and “What does this say about the participants’ positionality?” The thread was rich and engaging, and made us think about what role simulation could play in the EDIA context. We reflected to ourselves about how we were speaking about these issues, and about how deeply our socio-cultural, geographic, political, and our personal lenses were influencing our perceptions and actions. We could not tease out the complexities, given the deeply layered and intersectional forces at play. Clearly more questions were being raised than answers, and we felt motivated to explore together. We think that simulation is a great setting for doing so.

### The promises and perils of EDIA in simulation

Our underlying assumption is that making the world more diverse, providing access driven by principles of equity, and being more inclusive, are good things. We will be able to frame problems with more relevancy, find better solutions to those problems, and be clearer in evaluating whether our solutions have actually helped.

The healthcare simulation community has been exploring how to integrate principles of EDIA in its offerings [1–5], but there is room for more diversity and expanded scope. This is illustrated impressively in road safety, where women, elderly drivers, and drivers with larger bodies have higher risks of being injured in car accidents [6]. This has been linked to characteristics of crash test dummies, which by default represent standard male biodynamics, further leading to overgeneralization of safety findings [7]. While this is an important insight, we do know that simply diversifying mannikins in simulation scenarios does not go nearly far enough. Representation is an important step, but not a sufficient one. It might be tempting to stay there, but then we might have superficial diversity, where people (and simulators) with different characteristics are present together, without really engaging with each other on a deeper level. The careful use of language is one way of getting closer to deep diversity, and could be explored in simulation [8–10].

We urge the simulation community to reflect on how simulation can more expansively explore issues of EDIA. Collectively, our community appears to understand the need to reshape how health systems function, and that education can help to shift hearts and minds towards more just and fair healthcare (and eventually, we hope, fairer societies too). Healthcare simulation seeks to replicate aspects of our human world, and by extension, our complex healthcare environments. These structures influence and serve some individuals and groups more than others, and represent the differential economic, social, cultural, and psychological capital held in our societies. When we replicate ‘reality’ in simulation environments, we can modulate all kinds of elements of the simulated reality. We can create spaces to explore beyond just curiosity, and in fact, can confront how existing (power) structures can be dismantled and re-envisioned for more optimal redistribution of participation, power, and benefits. We can use simulation to experiment with variations of these realities, and start exploring consequences of such alternatives. Simulation is a suitable context in which to explore the multitude of layers of EDIA, as simulation combines opportunities for experience and reflection [11–14]. We argue that it is, in fact, our responsibility as simulation educators to take up this challenge, and to engage in meaningful scholarship to understand more about the impact of this work.

Like the discussion mentioned in the beginning of our editorial, we aim to surface tensions and complexities inherent in the pursuit of embedding EDIA in healthcare simulation, and to invite the community to explore these issues with us. How do we as a simulation community respond to this desire towards greater justice in healthcare? How do we resist the pressure for performative EDIA, instead pushing for training that challenges the deeply rooted structures that perpetuate harm and injustice? How can we avoid surface-level diversity that does not lead to any real changes? How can we consider what the potential perils are of addressing EDIA, which we may encounter if we do not approach this work carefully? In the current context, characterized by well-established hierarchies, inconsistent and differing terminology to describe these principles, and varied cultural norms, how do we come together as a community to bring about lasting change? How do we unite or balance different viewpoints in these matters? We invite our community’s exploration of the scholarship – both theoretical and empirical – required to deeply engage with these and many related questions.

Diversity, equity, and inclusion are identified in the literature as relevant for simulation and yet, there are no definitions of learning needs or broad explorations of topics in this area [15]. Our field has already done significant work in advancing discourse on the considerations and applications of simulation in exploring issues of structural racism and implicit bias [16–18]. Additionally, new scholarship and emerging training in this area now uses simulation as a tool for addressing racism in the workplace to promote learning, and perhaps more importantly, unlearning [19]. Simulation was used to help nursing students gauge their confidence in interviewing transgender patients and showed insecurities in the interaction with this group of patients [20]. The Canadian Alliance of Nurse Education Using Simulation provides a wealth of information and tools on how to teach and address 2SLGBTQ + topics with (and without) simulation [20]. In Lebanon, researcher and simulationist Zavi Lakissian works with LGBTQ + topics partly using simulation [21]. There are courses on care for transgender patients that are well received by participants [22].

Yet we cannot be complacent. It is also time to reflect about our own pratices in the simulation community more deeply. We might look at how genders, professional identities, race, age, among other identities and intersetionalities, show up across our simulation scenarios and within our simulation centres and programmes. How do we consider what co-creation and co-design look like with individuals and groups which may not be ‘easy’ or ‘convenient’ to engage with? This will require us to share our power as simulation designers, in order to create space and agency for others to tell us what is needed to accurately reflect their diverse and often generationally traumatic experiences and realities. Superficial diversity brings people with “diverse” backgrounds together. Deep diversity means taking on others’ points of view into a real and honest consideration [23]; this approach can also extend to discussions of deep equity, inclusion and accessibility.

## Conclusion

There are risks in this work. For simulation facilitators embracing EDIA, there may be ever present danger and vulnerability. The fear of saying the wrong thing, of making things worse, of being seen as ignorant or unaware of their own held biases, might keep many away from embedding EDIA principles in simulation practices more meaningfully and deeply. But we see so much potential in this lens, and some practical guidance is emerging on how to address these topics [7]. We need to keep going, and continue to explore and find new approaches, concepts, and practices that allow us to truly consider the interests, benefits, and diverse needs of patients, families, healthcare providers, and the broader healthcare system. We ask that you contribute to moving from the superficial consideration of EDIA in your work to a deeper one. Lean into the uncertainty, vulnerability and risks that this presents. Only then will we effect greater system change and social justice together.

## Data Availability

Not applicable.

## Abbreviations

EDIA: Used as an emcompassing term to describe issues and principles aligned with the pursuit of greater equity diversity inclusivity and accessibility. The authors acknowledge that these terms are vast and distinct individually and have been merely collapsed for the purpose of broader discussion in the commentary
LGBTQ+: A summary term to describe persons who are and/ or identify as lesbian, gay, bisexual, transgender, or queer. The plus indicates persons, who want see themselves included based on their gender-identity and / or sexual orientation
2LGBTQ+: A summary term to describe persons who are and/ or identify as two-spirited, lesbian, gay, bisexual, transgender, or queer. The plus indicates persons, who want see themselves included based on their gender-identity and / or sexual orientation
