# Correction: Moving towards deep equity, diversity, inclusivity and accessibility in simulation: a call to explore the promises and perils

**DOI:** 10.1186/s41077-025-00371-1

**Published:** 2025-08-18

**Authors:** Peter Dieckmann, Latika Nirula

**Affiliations:** 1 Copenhagen Academy for Medical Education and Simulation (CAMES), Herlev, Denmark Borgmester Ib Juuls Vej 1b, 2730; 2 Department of Quality and Health Technology, University in Stavanger, Stavanger, Norway; 3 Deparment of Public Health, Copenhagen University, Copenhagen, Denmark; 4 Li Ka Shing Knowledge Institute, Unity Health Toronto, Toronto, Canada; 5 Department of Psychiatry, University of Toronto, Toronto, Canada; 6 Centre for Faculty Development, University of Toronto, Toronto, Canada

**Correction: Adv Simul 9, 6 (2024)**

**https://doi.org/10.1186/s41077-024-00278-3**

In the original article [1], the following citation for the online discussion informing the questions raised in the Background section was omitted, and should be cited after the first sentence: https://x.com/StellaHPE/status/1535681104539271170?t=omqLKtaDjk3kWTsFA5x9cg&s=19.
